# Instantaneous Generation of Subject-Specific Finite Element Models of the Hip Capsule

**DOI:** 10.3390/bioengineering11010037

**Published:** 2023-12-28

**Authors:** Ahilan Anantha-Krishnan, Casey A. Myers, Clare K. Fitzpatrick, Chadd W. Clary

**Affiliations:** 1 Center of Orthopaedic Biomechanics, University of Denver, Denver, CO 80208, USA; ahilan.ananthakrishnan@du.edu (A.A.-K.); casey.myers@du.edu (C.A.M.); 2 Mechanical and Biomedical Engineering, Boise State University, Boise, ID 83725, USA; clarefitzpatrick@boisestate.edu

**Keywords:** subject-specific models, hip capsule, statistical shape model, surrogate modeling, total hip arthroplasty

## Abstract

Subject-specific hip capsule models could offer insights into impingement and dislocation risk when coupled with computer-aided surgery, but model calibration is time-consuming using traditional techniques. This study developed a framework for instantaneously generating subject-specific finite element (FE) capsule representations from regression models trained with a probabilistic approach. A validated FE model of the implanted hip capsule was evaluated probabilistically to generate a training dataset relating capsule geometry and material properties to hip laxity. Multivariate regression models were trained using 90% of trials to predict capsule properties based on hip laxity and attachment site information. The regression models were validated using the remaining 10% of the training set by comparing differences in hip laxity between the original trials and the regression-derived capsules. Root mean square errors (RMSEs) in laxity predictions ranged from 1.8° to 2.3°, depending on the type of laxity used in the training set. The RMSE, when predicting the laxity measured from five cadaveric specimens with total hip arthroplasty, was 4.5°. Model generation time was reduced from days to milliseconds. The results demonstrated the potential of regression-based training to instantaneously generate subject-specific FE models and have implications for integrating subject-specific capsule models into surgical planning software.

## 1. Introduction

Subject-specific alignment in total hip arthroplasty (THA) has emerged in recent years with the increased use of image-based planning, navigation, and robotic surgical systems [1,2]. Studies investigating the effectiveness of these systems demonstrate precise reconstruction of native hip anatomy, including implant positioning; fit with the host bone; and restoration of center-of-rotation, limb length, and offset [3,4]. These systems use bone-based imaging and three-dimensional (3D) templating software in preoperative planning to identify the optimal component size, angle, and position [5,6]. Furthermore, these systems offer intraoperative imaging to assist surgeons in performing precise bone resection and implant placement [7]. However, these systems do not include the hip capsule in their preoperative or intraoperative planning, which limits their ability to predict hip stability and range of motion after surgery.

Hip capsule function is complex, with each ligament structure in the capsule contributing to stability in certain degrees of freedom [8,9]. Studies report that reduced wrapping of the hip capsule around the smaller implanted femoral head after THA decreases its restraint to hip rotations [10,11]. Even though THA causes a loss of capsule function, there is a lack of consensus on the optimal surgical approach and capsule repair techniques to restore hip stability. For example, one group of studies reports no significant differences in dislocation risk between the anterior and posterior approach [12] or between the posterior and direct lateral approach [13]. Another cohort of studies reports significant differences in dislocation risk between direct anterior and posterior approaches [14] and between posterolateral and both direct anterior and anterolateral approaches [15]. Similarly, no clear consensus exists in capsular repair techniques, with some proponents for capsule repair [16,17] and others indifferent to the repair [18,19]. Thus, utilizing subject-specific models of the hip capsule to evaluate dislocation risk could reduce the cognitive burden of the surgeon and provide clarity on when capsule repair may be beneficial to prevent impingement.

**Figure 1:**
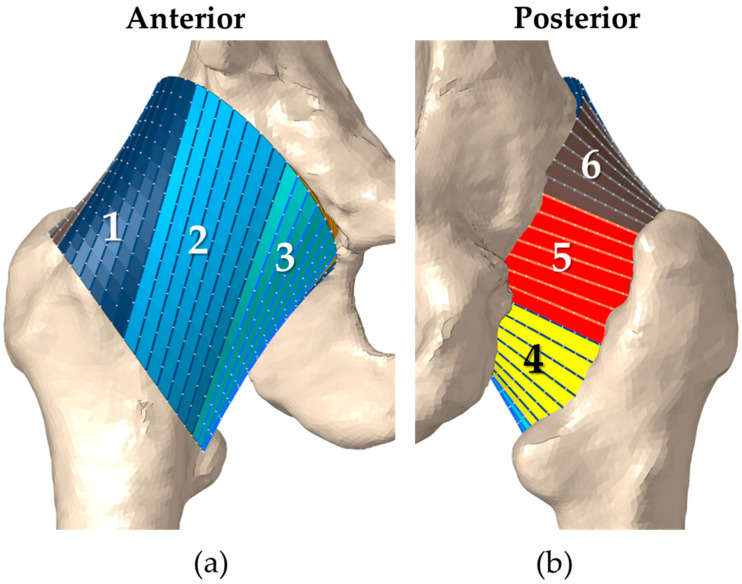
Anterior (**a**) and posterior (**b**) views of the implanted hip capsule were divided into six sectors, numbered circumferentially from the most superior attachment of the capsule.

A limited number of finite element hip capsule models have been reported in the literature. Elkins et al. developed a hip capsule model by tuning the material coefficients to match the load–displacement curve from a distraction test [20]. Myers et al. developed a probabilistic representation of the capsule by optimizing the ligament properties to match literature-reported average torque–rotation curves [21]. Our group recently developed subject-specific finite element capsule models which accurately predicted cadaveric hip torque–rotation responses (Figure 1) and validated the model predictions during movements known to cause dislocation [22]. These models accurately predict subject-specific laxity and, if integrated into the intraoperative workflow, could inform implant positioning and capsule repair.

Calibrating subject-specific models of the hip capsule to match experimental laxity from cadaveric specimens is time-intensive, requiring days to months depending on the number of material parameters, optimization algorithm, and processor speed [22,23]. This optimization time is untenable when the models are intended for intraoperative applications. Unfortunately, using a generalizable model formulated based on population-based means lacks the specificity in bone geometry, attachment sites, and ligament free length to accurately predict subject-specific laxity [22,24,25]. Practically, it is necessary to identify the minimum amount of laxity data required to calibrate capsule models with the fidelity necessary to detect clinically relevant differences. For instance, if the addition of a particular laxity to the training dataset only marginally improves the model’s prediction accuracy, it can be ignored, reducing the movements that must be measured in the clinic and the associated calibration time.

Statistical shape models are commonly used in orthopedic biomechanics to understand variation in bone morphology and to reconstruct 3D geometries from sparse image data [26,27]. A few studies have extended this technique to relate joint shapes with their function, resulting in statistical shape and function models. Examples include the relationship of knee anatomy with tibiofemoral and patellofemoral kinematics [28], total knee arthroplasty geometry and alignment with joint mechanics [29], and knee anatomy with knee laxity [30]. This paper aims to adapt shape–function statistical techniques to replace traditional calibration methods in the formulation of hip capsule models. Our hypothesis is that multilinear regression, relating the properties of the implanted hip capsule with hip laxity, can be used to instantaneously generate subject-specific FE models with equivalent accuracy to traditional optimization techniques, but in significantly less time. In addition, this study intends to identify relationships between the amount of input hip laxity (e.g., degrees of freedom and flexion angles) and the associated model accuracy.

## 2. Materials and Methods

### 2.1. Overview

**Figure 2:**
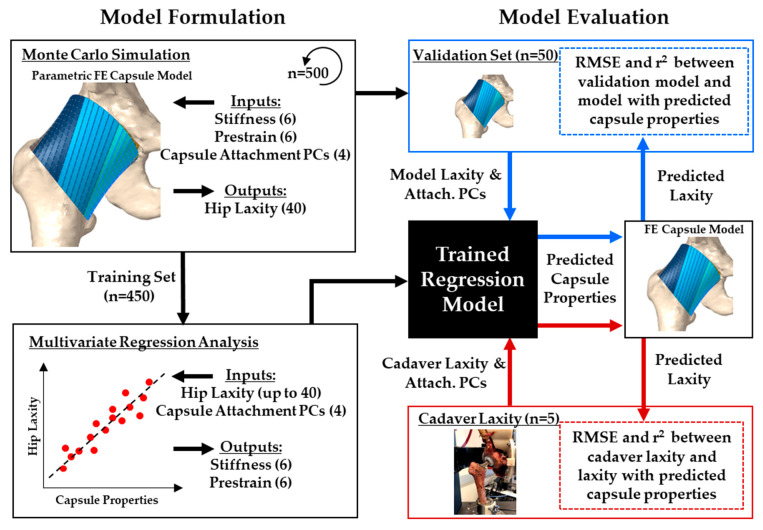
Workflow to develop the multilinear regression model. A training set of hip capsule models was generated from a validated FE model, including capsule properties, geometric variability, and the resulting hip laxity (**top left**). The training set was used to formulate a multilinear regression model that predicts capsule properties from hip laxity (**bottom left**). The regression model was tested through comparisons with FE models from the validation set (**top right**) and by predicting capsule properties for cadaveric hip specimen (**bottom right**).

A statistical framework to predict hip capsule parameters was developed based on a previously validated probabilistic finite element model of a cadaveric implanted hip capsule [22] (Figure 2). The base model was reconstructed from a single implanted cadaveric specimen, but the capsule was parameterized to recreate variability in attachment site geometry and mechanical properties. Variability in the capsule properties was based on the population of 10 previously reported cadaveric hip capsule specimens [22]. The parametric model was repeatedly evaluated via Monte Carlo simulations with Latin hypercube sampling to represent 95% of the population’s variability in capsule properties and attachment sites.

Multivariate regression models were developed that predict capsule properties (pre-strain and stiffness) from the subject’s hip laxity and attachment site geometry. The regression models were validated by predicting the hip capsule properties for five experimental cadaveric hips.

### 2.2. FE Model of the Implanted Hip Capsule

A previously validated specimen-specific implanted hip capsule model was used in the current study and is briefly described here [22]. Pelvis and femur bony geometry were segmented from a CT scan of a cadaveric hip implanted with a THA. The manufacturer’s implant CAD models (CORAIL^®^ Femoral Stems and PINNACLE^®^ modular dual mobility cups and liners, DePuy Synthes, Warsaw, IN, USA) were aligned with the implant geometries in the CT scan. The implants and bones were meshed using 3-noded 1.5 mm triangular elements (type S3R) and considered rigid.

The capsule was modeled as a cylindrical sleeve that originated from an ellipse around the acetabular rim and inserted into an ellipse around the femoral intertrochanteric line (Figure 1). An automated MATLAB script (MathWorks, Natick, MA, USA) generated a series of nodes connecting corresponding points on the pelvis and femur ellipses, wrapping around the femoral head when present. Corresponding nodes in adjacent lines were connected to form 4-noded quadrilateral elements along the length of the capsule. The capsule was subdivided into six longitudinal sectors to approximate the ligaments that compose the capsule. Each sector was modeled using fiber-reinforced quadrilateral hyperelastic membrane elements (type M3D4) with nonlinear tension-only springs embedded axially along the element edges (type CONN3D2) [31]. The stiffness and pre-strain of each sector were independently parameterized for sector-specific laxity definition and probabilistic analysis. The sector stiffness was defined as the total stiffness for the sector and divided across the number of spring elements acting in parallel in the sector mesh. Similarly, the sector pre-strain was equally divided among all springs acting in series along each longitudinal line of spring elements.

Contact between bones, implants, and the capsule was modeled using the general contact algorithm in Abaqus/Explicit, with all contact interactions considered rigid with the appropriate pressure–overclosure relationships to improve solver efficiency [32]. A coefficient of friction of 0.04 was applied at the cup–liner, head–liner, and stem–liner interfaces [33]. All other contacts (bone–bone, bone–implant, capsule–implant, and capsule–bone) were defined with a coefficient of friction of 0.01 [34]. In all trials, the pelvis was fixed in space, while 5 Nm torques were applied through the femur. The resulting hip rotations were calculated using passive connector elements embedded at the hip rotation center, using a three-cylindric open-chain configuration [35].

### 2.3. Statistical Shape Model of the Capsule

A principal component analysis (PCA) was performed on pelvis and femur bony geometry from 156 hips to quantify the population variability in the capsule attachment sites (39% female, age: 72 ± 11 years, height: 67 ± 3 in., weight: 147 ± 36 lbs). For each hip, the femur and pelvis bony geometry were manually segmented from high-resolution CT-scans (0.6 mm slice thickness). Anatomic landmarks were manually identified on the femur and pelvis and then used to reorient the bones into their respective anatomic coordinate systems. A coordinate system was assigned to the pelvis with the anterior–posterior (A-P) axis perpendicular to a plane defined by the anterior superior iliac spines and mid pubis point. For the femur, the superior–inferior axis was defined along the femoral mechanical axis and rotationally aligned with the femoral epicondylar axis in the transverse plane. The origins of the pelvis and femur coordinate systems were at the acetabular center and femoral head center, respectively.

**Figure 3:**
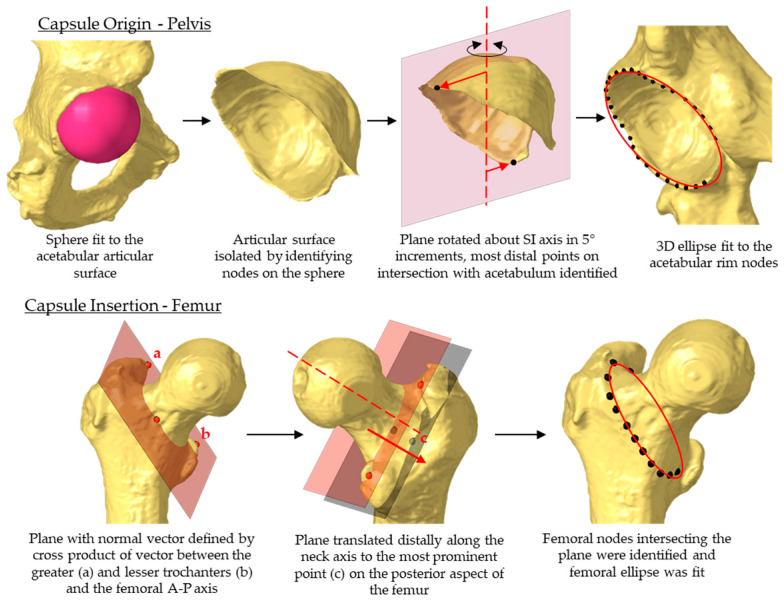
Automated workflow to identify origin and insertion sites of the hip capsule in a database of hip bony geometry.

The proximal end of the capsule was assumed to attach around the acetabular rim and was automatically extracted from the acetabular bone (Figure 3) [36,37]. Nodes were identified circumferentially along the apex of the acetabular rim, excluding the acetabular notch, and an ellipse was fit to the rim nodes. The femoral attachment of the capsule was assumed to follow the intertrochanteric crest [38]. The intertrochanteric crest was approximated by establishing a plane whose normal vector was defined as the cross product between a vector connecting the most prominent points on the greater and lesser trochanter and the *A-P* axis. The plane was translated distally along the femoral neck axis until it intersected the most prominent point on the posterior aspect of the femur [39]. The femur nodes intersecting this plane were extracted and fit with an ellipse.

To facilitate the PCA, 360 nodes were equally spaced along each ellipse, starting at the most superior point. PCA was performed on the 3D coordinates of the nodes [27]. The first four principal components (PCs) accounted for 77% of the capsule attachment site variability, and they were used to define the capsule geometry in subsequent probabilistic analyses (Table 1). New attachment geometries were reconstructed by multiplying PC scores with their corresponding PC vectors and adding these perturbations to the mean attachment coordinates.

### 2.4. Probabilistic Model Evaluation

A probabilistic analysis was performed on the FE model of the hip capsule, incorporating variability in both the capsule mechanical properties and attachment sites. The 500-trial Monte Carlo simulation used Latin hypercube sampling to select the capsule parameters from normal distributions (Table 2). The sixteen independent input variables included six capsule stiffnesses, six capsule pre-strains, and four PC scores for the capsule attachment sites. Each sample was selected from a normal distribution representing 95% of the population variability. The mean and standard deviations for the capsule mechanical properties (stiffness and pre-strain) were determined based on ten calibrated specimen-specific models from our previous study [22].

Isolated torques were applied to the femur in internal, external, abduction, and adduction rotations, while the hip was flexed to 0°, 30°, 45°, 60°, and 90° (20 total simulations). The applied torque followed a ramp-shaped waveform between 0 Nm and 5 Nm, starting from neutral femoral rotation [40]. The resulting rotations in each direction were recorded at 1 Nm of torque, corresponding to the slack-to-stiff transition of the capsule, and at 5 Nm torque. The probabilistic analysis resulted in a dataset of 500 independent trials relating 16 capsule properties to 40 laxity metrics and took 117 h of computation time to formulate (500 trials × ~14 min/trial).

### 2.5. Capsule Predictions Using Multilinear Regression

The 500-trial dataset was randomly divided, with 90% of trials (*n* = 450) being assigned to a training set and the remaining 10% being reserved for model validation (*n* = 50). From that data, multivariate regressions were performed. Inputs to the regression model were the 40 hip laxities (i.e., hip rotations at 1 Nm and 5 Nm) and the 4 PCs defining the capsule attachment sites. These variables were defined as inputs to the model because they could be measured either preoperatively (e.g., bony geometry from a CT scan for capsule attachments) or intraoperatively (e.g., hip laxity). The regression model outputs were the 12 capsule mechanical properties (stiffness and pre-strain for each capsule section). The multivariate regression took the following general form:

(1) $$p^{k}=\sum_{i=1}^{N}\left(l_{i}\times L_{i}\right)+\sum_{i=1}^{4}\left(p_{i}\times PC_{i}\right)$$

where *p^k^* represents the *k*th capsule parameter, *l_i_* represents the *i*th regression coefficient of the hip laxity parameters *L*, *p_i_* represents the *i*th regression coefficient of the attachment *PC* score, and *N* represents the number of laxity parameters used in the training set (Table 3). The regression coefficients were calculated using stepwise regression, which entails a systematic approach of adding and removing terms from a general linear model based on their statistical significance in explaining the response parameters (stepwiselm function, MATLAB, MathWorks, Natick, MA, USA).

**Figure 4:**
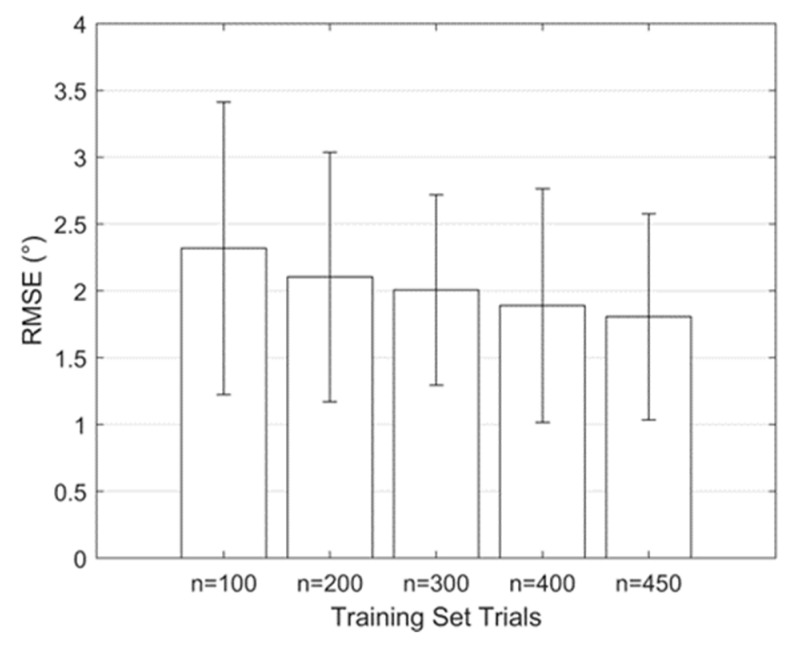
Convergence of the multivariate regression model where the cumulative RMSE decreased as the number of trials in the training set increased.

To check for convergence, five multivariate regression models were created based on training sets with 100, 200, 300, 400, and 450 trials. The regression models were used to predict the capsule mechanical parameters for each trial in the validation set (50 trials). FE models were then evaluated with the predicted capsule parameters and the known attachment site PCs to calculate the corresponding FE-model hip laxities. Root mean square errors (RMSEs) were calculated between the laxity predictions from the FE models and the corresponding laxity inputs to the regression models. Increasing the training set size from 400 to 450 trials resulted in a modest reduction in the overall RMSE and was thus considered converged (Figure 4). The full training set of 450 trials was used in all subsequent analyses.

The full training set included a comprehensive set of laxity measurements that may be burdensome to measure intraoperatively (i.e., hip laxity at 0°, 30°, 45°, 60°, and 90° flexion). Some of these laxity measurements may not be necessary to accurately predict the capsule’s mechanical properties. To evaluate the contribution of laxity measurement to the overall accuracy of the capsule predictions, regression models were trained using different combinations of laxity data. The baseline training set included internal–external (I-E) and adduction–abduction (Ad-Ab) at 0°, 30°, 60°, and 90° flexion. Variations in the training set included only training on I-E or Ad-Ab laxities and reducing the number of flexion angles to either 0°, 45° and 90°, or just 0° and 90° (Table 3). The model validation was repeated for each training set to calculate RMSEs between the input laxity and resulting FE-predicted laxity, using the capsule properties calibrated with the multilinear regression model. Student’s *t*-tests were performed to detect significant differences in RMSE between the baseline model and the 8 alternate training sets (*p* < 0.05).

### 2.6. Capsule Predictions for In Vitro Hip Laxity

The details of the experimental cadaveric hip laxity characterizations can be found in our previous study and are explained briefly here [22]: Five hips underwent THA, using the same dual mobility THA components evaluated in the FE model. Pre- and postoperative CT scans were taken to assess the anatomy and alignment of the bones and implants. The laxity of the hip capsules was experimentally characterized using a dynamic joint simulator (AMTI, Watertown, MA, USA). A motion capture system tracked the movements of the pelvis and femur during testing. Hip internal and external laxity were evaluated between 0 Nm and 5 Nm of torque at 0°, 30°, 60°, and 90° flexion.

The experimental data collection did not include laxity measurements at 45° flexion or Ad-Ab laxity measurements at any flexion angle, which were used in the formulation of some of the regression models described above. Thus, only the regression models trained on I-E laxity corresponding to the experiment were evaluated (Sets 2 and 8). Hip laxity and capsule attachment sites for the specimens were used to predict the capsule mechanical properties, using the two trained regression models. The predicted capsule parameters were evaluated in the specimen-specific FE models to evaluate the resulting hip laxity. RMSE and correlation coefficients (R^2^) between the measured and FE-model predicted hip laxities were calculated to evaluate the model performance.

## 3. Results

### 3.1. Shape Function Model of Hip Capsule and Probabilistic Model Response

**Figure 5:**
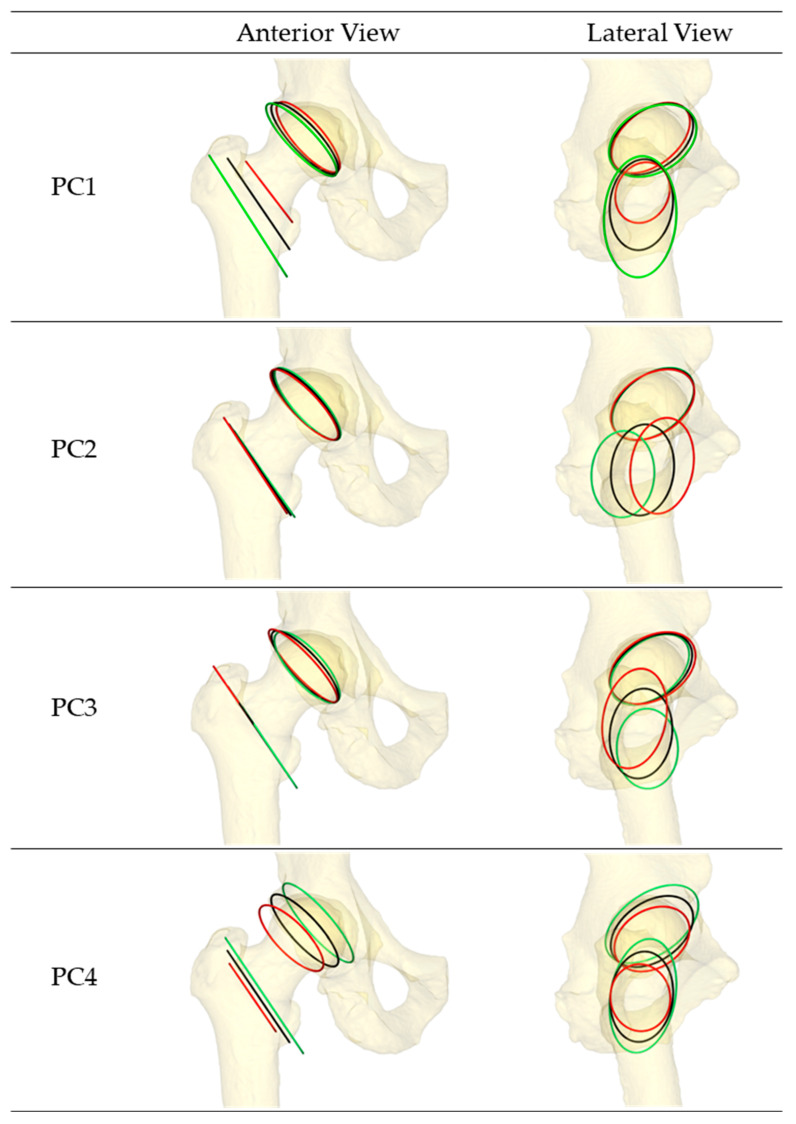
Origin and insertion of the capsule were approximated as ellipses to aid in parametrization and automation of the capsule creation. The black, red, and green lines represent mean, +2 standard deviations, and −2 standard deviations of PCs 1–4, respectively.

The first four modes of variation in the capsule attachment sites cumulatively accounted for 77% of the variability across the population (PC1: 36.8%, PC2: 18.0%, PC3: 13.0%, and PC4: 9.1%). When visualizing the attachment sites (Figure 5), the first PC corresponded to the length of the hip capsule (distance from the hip origin to the femoral attachment site) coupled with the superior–inferior length of the femoral attachment ellipse. PC2 described anterior–posterior translation of the intertrochanteric crest relative to the hip origin, corresponding to changes in native femoral version. PC3 accounted for variation in the superior–inferior position of the femoral attachment, corresponding to changes in femoral neck inclination. Finally, PC4 primarily described variation in the depth of the acetabulum coupled with changes in acetabular version.

**Figure 6:**
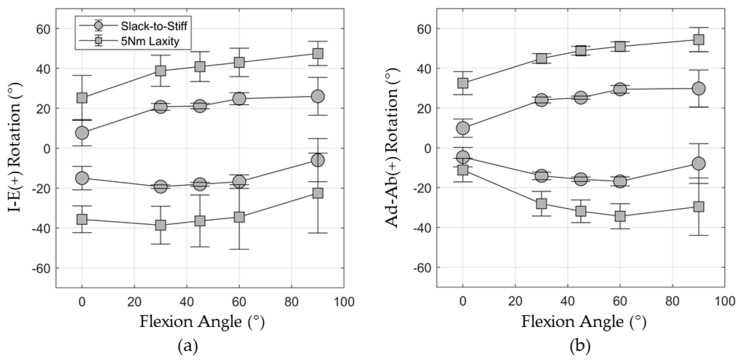
Probabilistic response of all 40 laxity parameters from the 500 trials during I-E rotation (**a**) and Ad-AB (**b**) rotations. Error bars show ±1 standard deviation.

The mean (±1 standard deviation) laxity response from the 500 probabilistic trials is reported in Figure 6. The mean external hip rotation increased from 25.2 ± 11.3° at 0° hip flexion to 47.5 ± 6.1° at 90° flexion under the 5 Nm torque. Conversely, internal hip rotation decreased from 35.7 ± 6.7° in extension to 22.5 ± 20.0° in 90° flexion. The highest overall I-E range of motion (77.5°) was observed at 60° hip flexion. Hip abduction laxity also increased with the increasing hip flexion, ranging from 32.5 ± 5.8° at 0° flexion to 54.4 ± 6.1° at 90° flexion. Similarly, the hip Ad-Ab range of motion was largest at 60° hip flexion (85.3°). The highest variability in the model’s laxity response was observed in deepest flexion for both Ad-Ab and I-E laxity. The internal rotation standard deviation was 20.0°, and the adduction rotation standard deviation was 14.4° at 90° hip flexion.

### 3.2. Regression Model Training, Validation, and Testing

The performance of the regression model improved when increasing the number of trials in the training set. The overall RMSE decreased from 2.3° ± 1.1° to 1.8° ± 0.8° when the training set increased from 100 to 450 trials (Figure 4). A percentage change in RMSE of <5% was considered an indicator of convergence, which was achieved with 450 trials (RMSE change of 4.2% between 400 trials and 450 trials). The average model training time for each set of laxity measures was ~720 s, while the model inference time was in the order of milliseconds. 

**Figure 7:**
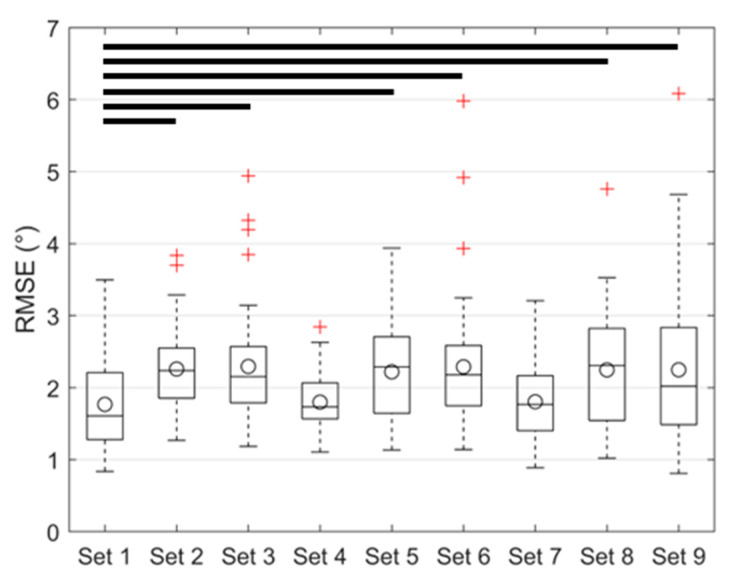
Box plot of RMSE when using different hip laxities in the training sets. Horizontal bars indicate statistical differences between sets (*p* < 0.05), circles indicate the mean RMSE, and + indicate outliers.

**Figure 8:**
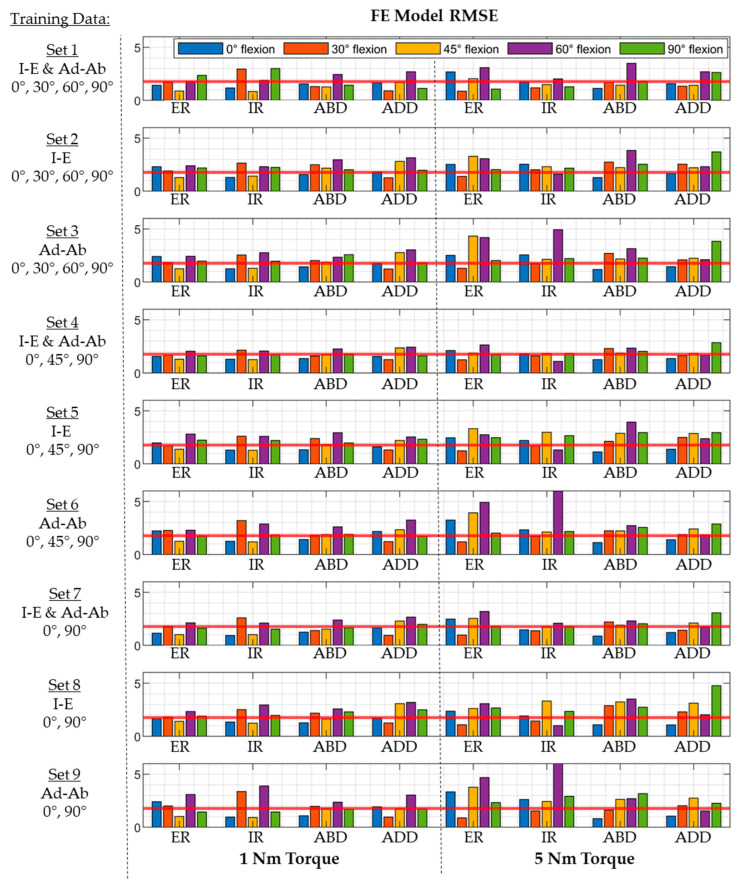
RMSE between FE models created using multilinear regression and test models from the initial probabilistic analysis for each combination of input laxity data. Horizontal red lines represent the mean RMSE across all 40 laxity measures.

The mean RMSE in laxity predictions varied depending on the types of laxity included in the training set (Figure 7 and Figure 8). The lowest composite RMSE of 1.8° ± 0.8° was achieved when all laxities were included in the training set (i.e., Set 1). The RMSE increased when trained on only Ad-Ab (2.3° ± 0.9°) or I-E (2.2° ± 0.6°) laxities at all flexion angles. Prediction accuracy was preserved when laxity at 45° hip flexion was used in lieu of 30° and 60° hip flexion (Set 4: 1.8° ± 0.4°). However, the accuracy was significantly reduced when either Ad-Ab or I-E laxities were excluded from the regression model (i.e., Training Sets 5 and 6). Model accuracy was likewise preserved when trained exclusively on hip laxity from 0° hip flexion to 90° hip flexion (Set 7, 1.8° ± 0.6°).

**Figure 9:**
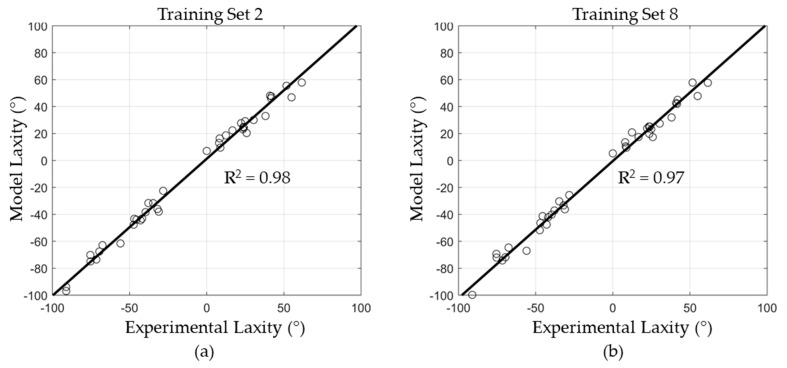
Correlation between experimental laxity and FE model predictions calibrated using multilinear regression, using Training Sets 2 (**a**) and 8 (**b**).

Prediction accuracy of the regression-based models was lower for the experimentally measured cadaveric hip laxity. The regression model trained on I-E laxity at all flexion angles (Set 2) predicted the cadaveric laxity with a RMSE of 4.5° ± 0.2° (range: 4.2° to 4.8°). The accuracy was reduced when the regression models were trained only on data from 0° and 90° hip flexion (Set 8): 4.8° ± 0.2° (range: 4.5° to 4.9°). Despite the higher RMSE compared to the validation cases, there were strong correlations between the model predicted and experimentally measured hip laxity with R^2^ = 0.98 and 0.97 for Training Sets 2 and 8, respectively (Figure 9). The FE models using capsule parameters predicted by the regression model performed slightly better than FE models developed using an optimization algorithm from our previous study, which had an average RMSE of 5.0° [22].

## 4. Discussion

The current study developed a statistical framework to calibrate hip capsule FE models that predict capsule laxity across a broad flexion range without the need for time-consuming optimization. RMSEs were less than 2° for calibrated capsules when predicting laxity generated by the probabilistic FE models (test set) and less than 5° for the cadaveric hip specimens.

The hip capsule resists hip dislocation in extreme ranges of motion [41]. Clinical studies have shown reduced dislocation rates and improved self-reported outcomes with the repair of the hip capsule [42,43]. However, other studies have demonstrated that most THA subjects have stable hips without capsule repair, particularly when using the direct anterior approach [44]. THA components with increased constraint, like lipped liners or dual-mobility constructs [45], or modularity between the stem and neck [46] can effectively mitigate the risk of dislocation, but the indications for use remain unclear. In most cases, constrained liners or dual-mobility constructs are reserved for revision surgeries [47]. Risk stratification tools for dislocation are primarily based on non-modifiable risk factors, like subject demographics, neurological disease, or previous spine surgery [48]. While directly responsible for resisting hip dislocation, the function of the hip capsule is rarely considered when evaluating dislocation risk in primary THA surgery. Incorporating computational models of the hip capsule into intraoperative planning software would provide surgeons with a quantitative method to assess the risk of dislocation and evaluate implant configurations and alignments to mitigate that risk.

Only a few studies have employed computational models to understand the hip capsule’s physiological structure–function relationship, evaluate surgical techniques for capsule management, or investigate THA implant contact mechanics. Elkins et al. developed a deterministic model of a single implanted hip capsule and calibrated the material properties to match an experimental load–distraction curve [20]. They subsequently used the model to parametrically evaluate the effects of changes in capsule thickness, attachment, incisions, and repairs on the capsule’s resistance to dislocation. Myers et al. developed a probabilistic representation of the implanted hip capsule and optimized the ligament properties to match the literature-reported average torque–rotation curves [21]. The average model was subsequently used in a parametric study to evaluate the effects of femoral stem offset, jump distance, and liner constraint on dislocation resistance [49]. Employing these same models for subject-specific surgical planning and intraoperative decision making is primarily limited by the computational resources and experimental data required to calibrate these models to represent each subject’s unique anatomy. The proposed statistical calibration in the current study directly addressed this prior limitation.

Surrogate statistical models trained using a limited number of finite element model evaluations have been used to provide computationally efficient predictions for several orthopedic applications. Surrogate modeling techniques include principal component regression [50], Bayesian statistics [51], artificial neural networks [52], and random forests [53]. Fitzpatrick et al. developed a regression-based surrogate model for predicting micromotion at the bone–implant interface in cementless total knee arthroplasty [54]. Their model achieved inference times of 30 s, compared to 15 h for traditional FE analysis when modeling the same boundary conditions. Ziaeipoor et al. used regression modeling to predict femoral strains during various activities of daily living [55] and reduced inference time from 66 s to 0.1 s with equivalent accuracy. Bartsoen et al. used artificial neural networks as surrogates for subject-specific musculoskeletal models of the knee [56]. The reduced inference time of the surrogate model enabled Bayesian optimization on large sets of ligament parameters for recreating subject-specific knee kinematics during a deep knee bend. This technique was extended to facilitate surgical planning by identifying implant alignments with the highest probability of restoring balance in the knee’s ligaments [57]. In the studies by Bartsoen et al., a unique neural network must be trained for every subject undergoing the planning process, which still requires significant computational time. Incorporating a statistical shape model of the hip capsule geometry into the multilinear regression developed in the current study allowed the same regression model to be applied across the patient population, improving the generalizability in surgical settings.

Population-level probabilistic studies predicting ligament behavior generally exclude ligament attachment site variability from the parameter space, as this necessitates a large database of anatomical geometries and additional model preprocessing. Statistical shape models of the femur and pelvis have become common in recent years; however, models combining the three-dimensional geometry of both bones into a single model are less so. Zhang et al. analyzed the three-dimensional shape of native femurs and found that the first three modes of variation corresponded to femur scale, femur version, and neck inclination, respectively [39]. These results were consistent with the SSM in the current study, accounting for variation in the femoral capsular attachments. Studies of pelvis morphology have not found variation in the acetabular geometry (i.e., version and inclination) among the common modes of pelvis variability [58,59]. The current SSM likewise observes minimal variation in acetabular inclination among the dominant shape modes and decreased diameter of the acetabular rim coupled with decreased acetabular version as part of mode 4 (Figure 5 and Table 1).

Different combinations of hip laxity were used as training data to quantify the number of laxity measurements needed for accurate model calibration. The results demonstrated that including hip laxity measured in mid-flexion (i.e., 30° and 60° or 45°) did not result in meaningful improvements to the prediction accuracy. A previous analysis of capsular recruitment found that all sectors of the capsule were activated during either I-E or Ad-Ab rotations at 0° and 90° flexion. Specifically, sectors 1, 2, and 6 were recruited during internal rotation, while sectors 1, 4, 5, and 6 were recruited during external rotation. Similarly, sectors 1–4 were recruited during abduction, while sectors 1–3 were recruited during adduction [22,60]. Given that all six capsule sectors were activated during this subset of hip rotations, there was sufficient information contained in the laxity to accurately predict capsule properties. When either Ad-Ab or I-E laxities were removed from the training data, the RMSE was statistically higher than the baseline model (Set 1, *p* < 0.05; Figure 7). Models trained only on I-E laxity had larger errors in the Ad-Ab degree of freedom, and vice versa (Figure 8). However, these increases were relatively small, and these models may still have sufficient accuracy depending on the clinical application.

The model calibration approach described in this study focused on calibration of hips implanted with THA. This simplification negated consideration of the native femoral head geometry and potential variations in the implant positioning in the model formulation. To facilitate surgical planning, the approach must be extended to calibrate capsule properties in native hips, incorporating the native femoral head anatomy. Future work will focus on collecting hip laxity in native hips, the calibration of native hip capsule models, and demonstrating the accuracy of these models for predicting changes in hip stability resulting from THA surgery.

Additional limitations in the current study include simplifications to the FE hip capsule model to facilitate probabilistic analysis, including assuming a cylindrical geometry for the capsule ligaments with ellipsoidal attachment sites, modeling the capsule with fiber-reinforced membrane elements, and dividing the capsule into six arbitrary sectors. Simplifications in the model formulation are evidenced by the higher RMSE when predicting cadaveric laxity compared to FE-generated laxity from models in the test set. However, the predictive accuracy of the current capsule representations was equivalent to previous capsule representations that modeled individual capsule structures [21,22], indicating that the simplification did not significantly reduce prediction accuracies. This may, however, limit the ability of the model to accurately predict capsule strains or changes in capsule function associated with capsular incisions or releases. The translation of these models into planning applications also requires a hip laxity assessment, using methods available in the operating room, potentially leveraging instrument surgical tables (e.g., Hana tables) and clinical evaluations like axial distraction (i.e., shuck test).

## 5. Conclusions

Management of the hip capsule during THA and determining the optimal implant configuration to prevent dislocation are common challenges faced by orthopedic surgeons. Computational subject-specific hip capsule models could be used to provide quantitative stability metrics for use during intraoperative planning and robotic surgery. The current study demonstrated a statistical method coupling a probabilistic FE model with multilinear regression that instantaneously generates calibrated subject-specific models. The calibrated models achieved RMSEs less than 2° when predicting FE-generated hip laxity and less than 5° when predicting hip laxity measured in cadavers. The methods demonstrated in this study enable future work towards translating computational models into valuable intraoperative planning tools for THA.

## Figures and Tables

**Table 1:** Orientation of the attachment site ellipses with respect to the global axes.

PC (Variance Explained)		Acetabulum (Origin)		Femur (Insertion)	
		Inclination (°)	Version (°)	Inclination (°)	Version (°)
Mean		137.9	18.5	124.1	0.0
PC1 (36.8%)	+2 SD	138.5	20.7	127.2	0.0
	−2 SD	137.5	16.6	122.5	0.0
PC2 (18.0%)	+2 SD	138.0	17.7	123.3	0.0
	−2 SD	137.8	19.4	125.0	0.0
PC3 (13.0%)	+2 SD	136.6	14.4	124.4	0.0
	−2 SD	138.9	22.7	123.9	0.0
PC4 (9.1%)	+2 SD	137.5	21.7	124.4	0.0
	−2 SD	137.4	15.6	124.0	0.0

**Table 2:** Input parameter distributions in the probabilistic model.

Parameter ID	Parameter	Mean ± Standard Deviation
1	Sector 1—Stiffness (N/mm)	61.0 ± 15.0
2	Sector 2—Stiffness (N/mm)	62.3 ± 15.0
3	Sector 3—Stiffness (N/mm)	58.5 ± 15.0
4	Sector 4—Stiffness (N/mm)	53.9 ± 15.0
5	Sector 5—Stiffness (N/mm)	70.5 ± 15.0
6	Sector 6—Stiffness (N/mm)	50.4 ± 15.0
7	Sector 1—Pre-strain	0.99 ± 0.05
8	Sector 2—Pre-strain	0.71 ± 0.05
9	Sector 3—Pre-strain	0.62 ± 0.05
10	Sector 4—Pre-strain	0.57 ± 0.05
11	Sector 5—Pre-strain	0.48 ± 0.05
12	Sector 6—Pre-strain	0.57 ± 0.05
13	Attachment Region PC1	±2.0
14	Attachment Region PC2	±2.0
15	Attachment Region PC3	±2.0
16	Attachment Region PC4	±2.0

**Table 3:** Different sets of laxity parameters were used in the training set to identify the minimal number (#) of parameters required to achieve accuracy comparable to the baseline model.

Training Set	Laxity the Training Set	Hip Flexion (Deg)	# Input Parameters (N)
Set 1	I-E laxity and Ad-Ab laxity	0, 30, 60, 90	32
Set 2	I-E laxity	0, 30, 60, 90	16
Set 3	Ad-Ab laxity	0, 30, 60, 90	16
Set 4	I-E Laxity and Ad-Ab laxity	0, 45, 90	24
Set 5	I-E laxity	0, 45, 90	12
Set 6	Ad-Ab laxity	0, 45, 90	12
Set 7	I-E laxity and Ad-Ab laxity	0, 90	16
Set 8	I-E laxity	0, 90	8
Set 9	Ad-Ab laxity	0, 90	8

## Data Availability

Data available upon request from the corresponding author.

## References

[1] Colombi A., Schena D., Castelli C.C. (2019). Total hip arthroplasty planning. EFORT Open Rev..

[2] Subramanian P., Wainwright T.W., Bahadori S., Middleton R.G. (2019). A review of the evolution of robotic-assisted total hip arthroplasty. Hip Int..

[3] Ng N., Gaston P., Simpson P.M., Macpherson G.J., Patton J.T., Clement N.D. (2021). Robotic arm-assisted versus manual total hip arthroplasty: A systematic review and meta-analysis. Bone Jt. J..

[4] Emara A.K., Samuel L.T., Acuña A.J., Kuo A., Khlopas A., Kamath A.F. (2021). Robotic-arm assisted versus manual total hip arthroplasty: Systematic review and meta-analysis of radiographic accuracy. Int. J. Med. Robot. Comput. Assist. Surg..

[5] Riddick A., Smith A., Thomas D.P. (2014). Accuracy of preoperative templating in total hip arthroplasty. J. Orthop. Surg..

[6] Jacofsky D.J., Allen M. (2016). Robotics in arthroplasty: A comprehensive review. J. Arthroplast..

[7] Fontalis A., Kayani B., Thompson J.W., Plastow R., Haddad F.S. (2022). Robotic total hip arthroplasty: Past, present and future. Orthop. Trauma.

[8] Martin H.D., Savage A., Braly B.A., Palmer I.J., Beall D.P., Kelly B. (2008). The function of the hip capsular ligaments: A quantitative report. Arthrosc. J. Arthrosc. Relat. Surg..

[9] Ng K.G., Jeffers J.R., Beaulé P.E. (2019). Hip joint capsular anatomy, mechanics, and surgical management. J. Bone Jt. Surg. Am. Vol..

[10] van Arkel R.J., Ng K.G., Muirhead-Allwood S.K., Jeffers J.R. (2018). Capsular ligament function after total hip arthroplasty. The Journal of Bone and Joint surgery. Am. Vol..

[11] Logishetty K., Van Arkel R.J., Ng K.C.G., Muirhead-Allwood S.K., Cobb J.P., Jeffers J.R.T. (2019). Hip capsule biomechanics after arthroplasty: The effect of implant, approach, and surgical repair. Bone Jt. J..

[12] Maratt J.D., Gagnier J.J., Butler P.D., Hallstrom B.R., Urquhart A.G., Roberts K.C. (2016). No difference in dis-location seen in anterior vs posterior approach total hip arthroplasty. J. Arthroplast..

[13] Mjaaland K.E., Svenningsen S., Fenstad A.M., Havelin L.I., Furnes O., Nordsletten L. (2017). Implant survival after minimally invasive anterior or anterolateral vs. conventional posterior or direct lateral approach: An analysis of 21,860 total hip arthroplasties from the Norwegian Arthroplasty Register (2008 to 2013). J. Bone Jt. Surg..

[14] Haynes J.A., Hopper R.H., Ho H., McDonald III J.F., Parks N.L., Hamilton W.G. (2022). Direct anterior approach for primary total hip arthroplasty lowers the risk of dislocation compared to the posterior approach: A single institution experience. J. Arthroplast..

[15] Zijlstra W.P., De Hartog B., Van Steenbergen L.N., Scheurs B.W., Nelissen R.G. (2017). Effect of femoral head size and surgical approach on risk of revision for dislocation after total hip arthroplasty: An analysis of 166,231 procedures in the Dutch Arthroplasty Register (LROI). Acta Orthop..

[16] Swanson T.V., Kukreja M.M., Ballard J.C., Calleja H.G., Brown J.M. (2019). The “capsular noose”: A new capsular repair technique to diminish dislocation risk after the posterior approach total hip arthroplasty. Int. J. Surg. Open.

[17] Pedneault C., Tanzer D., Nooh A., Smith K., Tanzer M. (2020). Capsular closure outweighs head size in preventing dislocation following revision total hip arthroplasty. HIP Int..

[18] Vandeputte F.J., Vanbiervliet J., Sarac C., Driesen R., Corten K. (2021). Capsular resection versus capsular repair in direct anterior approach for total hip arthroplasty: A randomized controlled trial. Bone Jt. J..

[19] Schwartz A.M., Goel R.K., Sweeney A.P., Bradbury T.L. (2021). Capsular management in direct anterior total hip arthroplasty: A randomized, single-blind, controlled trial. J. Arthroplast..

[20] Elkins J.M., Stroud N.J., Rudert M.J., Tochigi Y., Pedersen D.R., Ellis B.J., Brown T.D. (2011). The capsule’s contribution to total hip construct stability–a finite element analysis. J. Orthop. Res..

[21] Myers C.A., Fitzpatrick C.K., Huff D.N., Laz P.J., Rullkoetter P.J. (2020). Development and calibration of a probabilistic finite element hip capsule representation. Comput. Methods Biomech. Biomed. Eng..

[22] Anantha Krishnan A., Myers C.A., Scinto M., Marshall B.N., Clary C.W. (2023). Specimen-specific finite element representations of implanted hip capsules. Comput. Methods Biomech. Biomed. Eng..

[23] Ezquerro F., Vacas F.G., Postigo S., Prado M., Simón A. (2011). Calibration of the finite element model of a lumbar functional spinal unit using an optimization technique based on differential evolution. Med. Eng. Phys..

[24] Ewing J.A., Kaufman M.K., Hutter E.E., Granger J.F., Beal M.D., Piazza S.J., Siston R.A. (2016). Estimating subject-specific soft-tissue properties in a TKA knee. J. Orthop. Res..

[25] Bischoff J.E., Dai Y., Goodlett C., Davis B., Bandi M. (2014). Incorporating population-level variability in orthopedic biomechanical analysis: A review. J. Biomech. Eng..

[26] Patil A., Kulkarni K., Xie S., Bull A.M., Jones G.G. (2023). The accuracy of statistical shape models in predicting bone shape: A systematic review. Int. J. Med. Robot. Comput. Assist. Surg..

[27] Sarkalkan N., Weinans H., Zadpoor A.A. (2014). Statistical shape and appearance models of bones. Bone.

[28] Smoger L.M., Fitzpatrick C.K., Clary C.W., Cyr A.J., Maletsky L.P., Rullkoetter P.J., Laz P.J. (2015). Statistical modeling to characterize relationships between knee anatomy and kinematics. J. Orthop. Res..

[29] Gibbons K.D., Clary C.W., Rullkoetter P.J., Fitzpatrick C.K. (2019). Development of a statistical shape-function model of the implanted knee for real-time prediction of joint mechanics. J. Biomech..

[30] Shalhoub S., Cyr A., Maletsky L.P. (2022). Correlation between knee anatomy and joint laxity using principal component analysis. J. Orthop. Res..

[31] Baldwin M.A., Clary C.W., Fitzpatrick C.K., Deacy J.S., Maletsky L.P., Rullkoetter P.J. (2012). Dynamic finite element knee simulation for evaluation of knee replacement mechanics. J. Biomech..

[32] Fitzpatrick C., Baldwin M., Rullkoetter P. (2010). Computationally Efficient Finite Element Evaluation of Natural Patellofemoral Mechanics. J. Biomech. Eng..

[33] Brockett C., Williams S., Jin Z., Isaac G., Fisher J. (2007). Friction of total hip replacements with different bearings and loading conditions. J. Biomed. Mater. Res. Part B Appl. Biomater..

[34] Guezmil M., Bensalah W., Mezlini S. (2016). Tribological behavior of UHMWPE against TiAl6V4 and CoCr28Mo alloys under dry and lubricated conditions. J. Mech. Behav. Biomed. Mater..

[35] Grood E.S., Suntay W.J. (1983). A joint coordinate system for the clinical description of three-dimensional motions: Application to the knee. J. Biomech. Eng..

[36] Telleria J.J., Lindsey D.P., Giori N.J., Safran M.R. (2014). A quantitative assessment of the insertional footprints of the hip joint capsular ligaments and their spanning fibers for reconstruction. Clin. Anat..

[37] Tsutsumi M., Nimura A., Honda E., Utsunomiya H., Uchida S., Akita K. (2019). An anatomical study of the ante-rosuperior capsular attachment site on the acetabulum. J. Bone Jt. Surgery. Am. Vol..

[38] Wingstrand H., Wingstrand A. (1997). Biomechanics of the hip joint capsule—A mathematical model and clinical implications. Clin. Biomech. (Bristol Avon).

[39] Zhang J., Malcolm D., Hislop-Jambrich J., Thomas C.D.L., Nielsen P.M. (2014). An anatomical region-based sta-tistical shape model of the human femur. Comput. Methods Biomech. Biomed. Eng. Imaging Vis..

[40] van Arkel R.J., Amis A.A., Jeffers J.R. (2015). The envelope of passive motion allowed by the capsular ligaments of the hip. J. Biomech..

[41] Karunaseelan K.J., Dandridge O., Muirhead-Allwood S.K., van Arkel R.J., Jeffers J.R. (2021). Capsular ligaments provide a passive stabilizing force to protect the hip against edge loading. Bone Jt. Res..

[42] Sun X., Zhu X., Zeng Y., Zhang H., Zeng J., Feng W., Zeng Y. (2020). The effect of posterior capsule repair in total hip arthroplasty: A systematic review and meta-analysis. BMC Musculoskelet. Disord..

[43] Owens J.S., Jimenez A.E., Shapira J., Saks B.R., Glein R.M., Maldonado D.R., Domb B.G. (2021). Capsular repair may improve outcomes in subjects undergoing hip arthroscopy for femoroacetabular impingement: A systematic review of comparative outcome studies. Arthrosc. J. Arthrosc. Relat. Surg..

[44] Batailler C., Fary C., Verdier R., Aslanian T., Caton J., Lustig S. (2017). The evolution of outcomes and indications for the dual-mobility cup: A systematic review. Int. Orthop..

[45] Grace T.R., Goh G.S., Lee G.C., Kamath A.F., Kurtz S.M., Courtney P.M. (2021). Dual Mobility Reduces Dislocations—Why I Use It in All Revisions. J. Arthroplast..

[46] Zajc J., Fokter S.K. (2023). Bimodular femoral stems in primary total hip arthroplasty. Expert Rev. Med. Devices.

[47] Vajapey S.P., Fideler K.L., Lynch D., Li M. (2020). Use of dual mobility components in total hip arthroplasty: Indications and outcomes. J. Clin. Orthop. Trauma.

[48] Wyles C.C., Maradit-Kremers H., Larson D.R., Lewallen D.G., Taunton M.J., Trousdale R.T., Sierra R.J. (2022). Creation of a total hip arthroplasty subject-specific dislocation risk calculator. J. Bone Jt. Surg..

[49] Myers C.A., Huff D.N., Mason J.B., Rullkoetter P.J. (2022). Effect of intraoperative treatment options on hip joint stability following total hip arthroplasty. J. Orthop. Res. Off. Publ. Orthop. Re-Search Soc..

[50] Fitzpatrick C.K., Baldwin M.A., Rullkoetter P.J., Laz P.J. (2011). Combined probabilistic and principal component analysis approach for multivariate sensitivity evaluation and application to implanted patellofemoral mechanics. J. Biomech..

[51] Bah M.T., Nair P.B., Taylor M., Browne M. (2011). Efficient computational method for assessing the effects of implant positioning in cementless total hip replacements. J. Biomech..

[52] Taylor M., Perilli E., Martelli S. (2017). Development of a surrogate model based on subject weight, bone mass and geometry to predict femoral neck strains and fracture loads. J. Biomech..

[53] Donaldson F.E., Nyman E., Coburn J.C. (2015). Prediction of contact mechanics in metal-on-metal Total Hip Re-placement for parametrically comprehensive designs and loads. J. Biomech..

[54] Fitzpatrick C.K., Hemelaar P., Taylor M. (2014). Computationally efficient prediction of bone–implant interface micromotion of a cementless tibial tray during gait. J. Biomech..

[55] Ziaeipoor H., Martelli S., Pandy M., Taylor M. (2019). Efficacy and efficiency of multivariate linear regression for rapid prediction of femoral strain fields during activity. Med. Eng. Phys..

[56] Bartsoen L., Faes M.G., Andersen M.S., Wirix-Speetjens R., Moens D., Jonkers I., Vander Sloten J. (2023). Bayesian parameter estimation of ligament properties based on tibio-femoral kinematics during squatting. Mech. Syst. Signal Process..

[57] Bartsoen L., Faes M.G.R., Wirix-Speetjens R., Moens D., Jonkers I., Sloten J.V. (2022). Probabilistic planning for ligament-balanced TKA-Identification of critical ligament properties. Front. Bioeng. Biotechnol..

[58] Brynskog E., Iraeus J., Reed M.P., Davidsson J. (2021). Predicting pelvis geometry using a morphometric model with overall anthropometric variables. J. Biomech..

[59] van Veldhuizen W.A., van der Wel H., Kuipers H.Y., Kraeima J., Ten Duis K., Wolterink J.M., IJpma F.F. (2023). Development of a Statistical Shape Model and Assessment of Anatomical Shape Variations in the Hemipelvis. J. Clin. Med..

[60] van Arkel R.J., Amis A.A., Cobb J.P., Jeffers J.R.T. (2015). The capsular ligaments provide more hip rotational re-straint than the acetabular labrum and the ligamentum teres: An experimental study. Bone Jt. J..

